# A Rare Case of a Complex Nabothian Cyst in the Cervix: A Diagnostic Conundrum

**DOI:** 10.7759/cureus.92195

**Published:** 2025-09-13

**Authors:** Rohit Chakravarty, Nilanjan Sarkar, Sandipan Mukhopadhyay

**Affiliations:** 1 Radiology, Tata Main Hospital, Jamshedpur, IND

**Keywords:** cervical mass, complex nabothian cyst, magnetic resonance imaging, multiloculated, septated

## Abstract

Nabothian cysts are benign cervical cysts commonly found in women of reproductive age. Typically small in size, they often do not exhibit clinical symptoms. These cysts usually resolve spontaneously without the need for medical intervention. In rare cases, however, they can enlarge significantly, potentially compressing surrounding organs and leading to symptoms associated with the mass effect.

A 46-year-old female patient presented to our hospital for a routine health checkup. During the examination, an incidental finding of a cervical mass was detected on a pelvic ultrasound. Further imaging of the cervical mass was performed using magnetic resonance imaging (MRI), which confirmed the presence of a large, multiloculated, and septated complex Nabothian cyst. This case report illustrates the asymptomatic nature of complex Nabothian cysts and emphasizes the efficacy of MRI as a crucial diagnostic tool for identifying pelvic lesions.

## Introduction

Nabothian cysts are small cystic lesions located in the cervix and are typically asymptomatic. However, in rare cases, they may enlarge and become symptomatic due to the compression of adjacent soft tissue [1]. Ultrasound is the first-line imaging modality for visualizing female pelvic anatomy and pathology [2]. Computed tomography is not considered an effective imaging modality for assessing female pelvic pathology, as it does not provide sufficient soft tissue contrast [3,4]. In contrast, magnetic resonance imaging (MRI) offers superior visualization of anatomy and cervical lesions due to its enhanced soft tissue contrast [2-4]. This case report describes a large, multiloculated, and septated Nabothian cyst in an asymptomatic patient who was undergoing a routine ultrasound checkup. MRI facilitated the accurate diagnosis of the cystic lesion in the cervix.

## Case presentation

**Figure 1:**
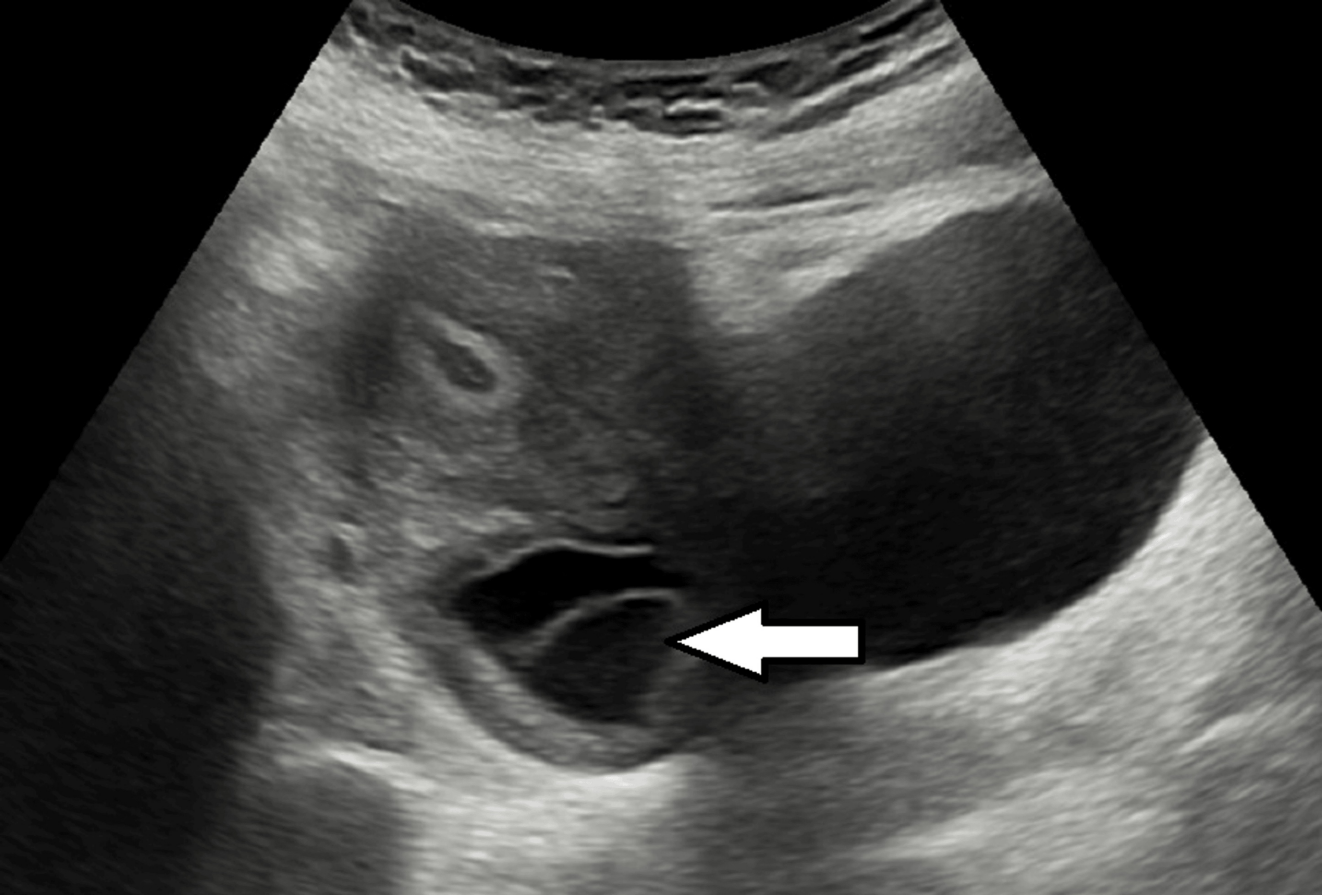
Ultrasound of the cervix showing a multiseptated cystic lesion.

A 46-year-old female patient visited our institution for a routine checkup and underwent an ultrasound of the abdomen and pelvis. Her pulse was regular and within the normal range. Blood pressure in the sitting position was also within normal limits. Routine hematological examinations yielded normal results. The ultrasound of the upper abdomen appeared normal; however, an incidental finding of a septated cystic lesion in the pelvis was noted, measuring approximately 5 x 4 cm (Figure 1). Subsequent physical examination revealed an enlarged cervix with multiple palpable cystic structures, completely occluding the os. A pap smear was performed. It was negative for intraepithelial lesions or malignancy, providing reassurance but not fully addressing the nature of the cysts. Therefore, an MRI of the pelvis was recommended for further evaluation.

**Figure 2:**
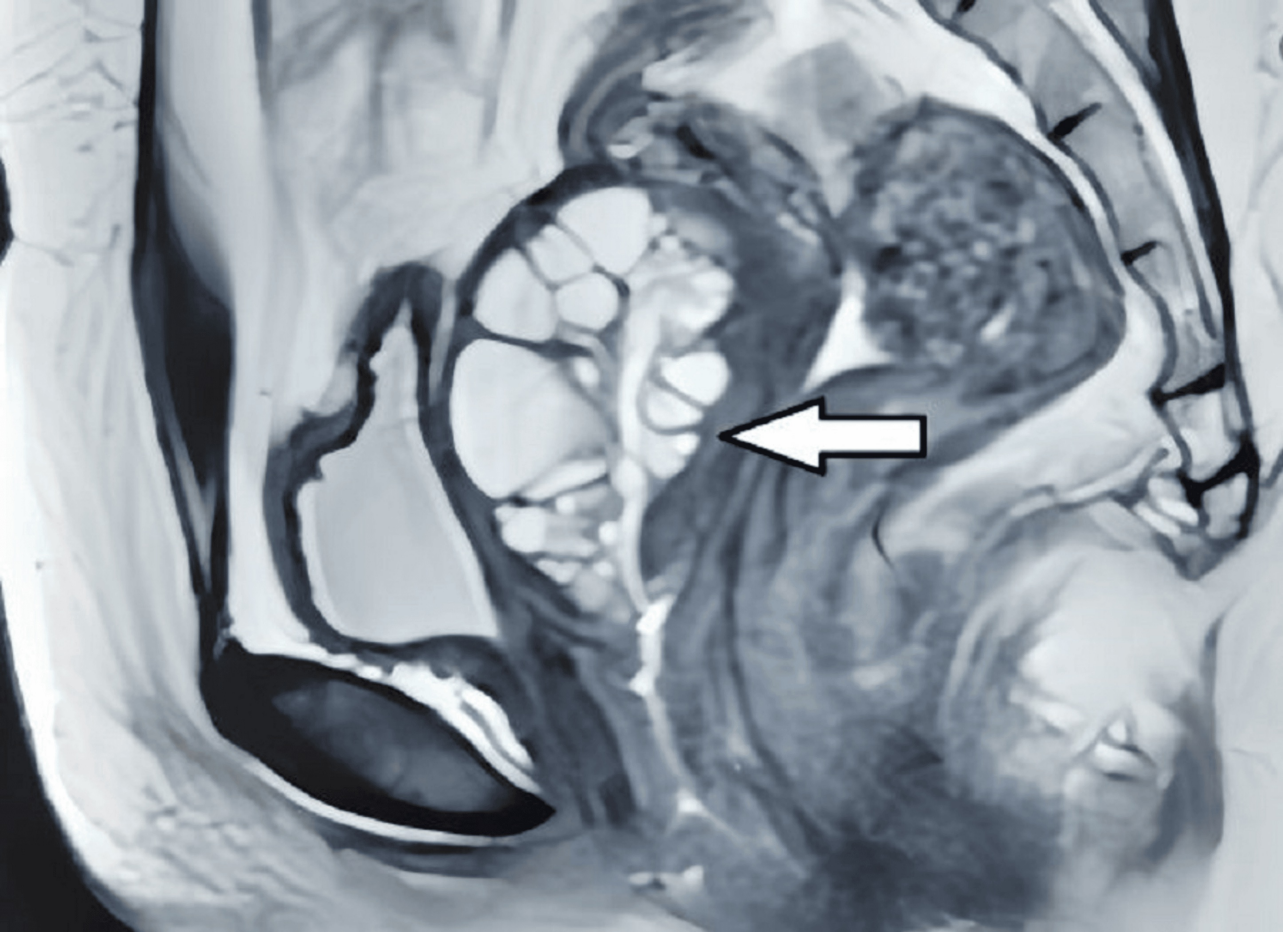
T2-weighted MRI showing the sagittal section of the pelvis. A hyperintense septated cystic lesion involving the cervix is noted.

**Figure 3:**
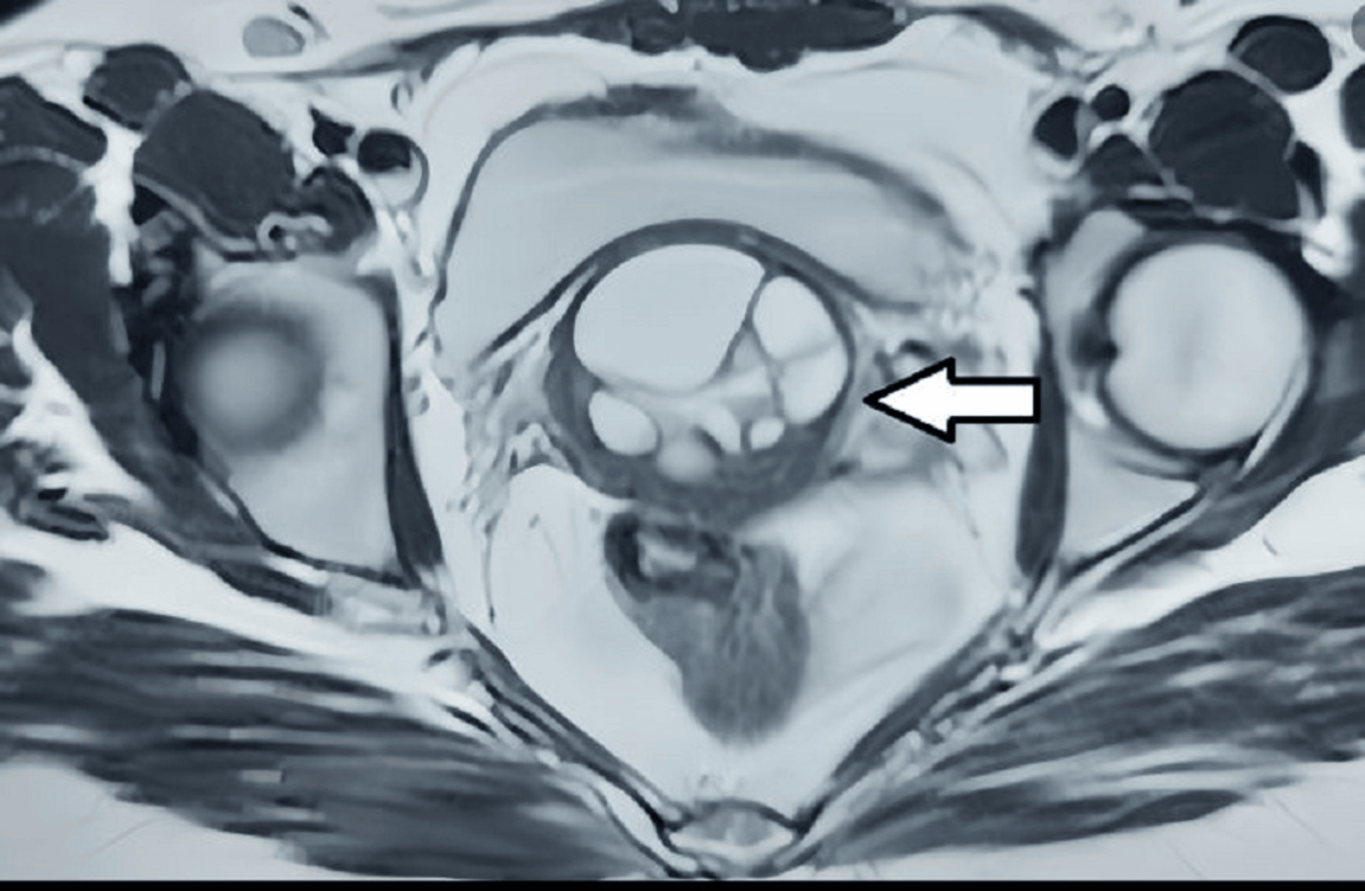
T2-weighted MRI showing the axial section of the pelvis. A hyperintense septated cystic lesion involving the cervix is noted. No involvement of the parametrium is noted.

**Figure 4:**
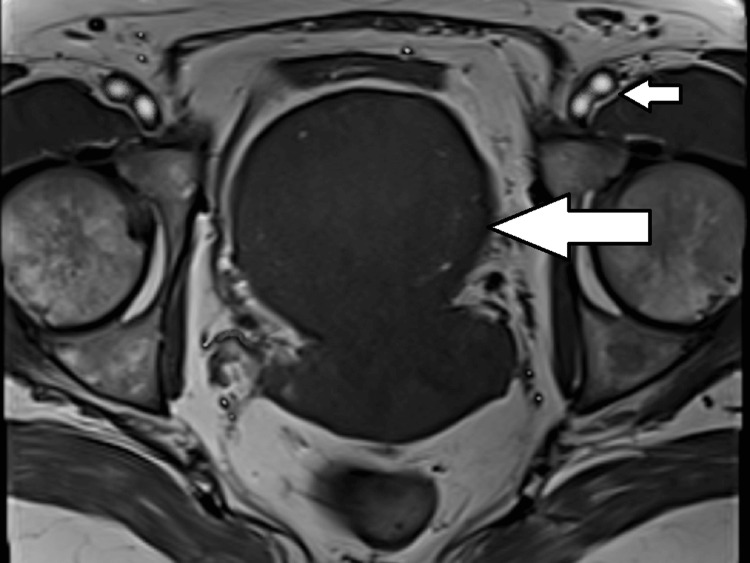
Post contrast T1-weighted axial section of the pelvis shows no enhancement of the lesion (large arrow) in comparison to the enhancing iliac vessels (small arrow).

**Figure 5:**
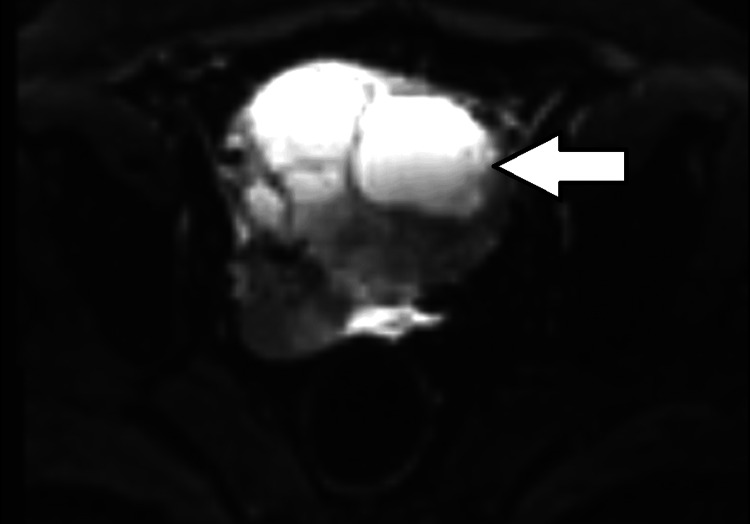
DW (diffusion weighted) image of the cystic lesion of the cervix showing a bright cystic lesion with septations. No solid component is seen.

**Figure 6:**
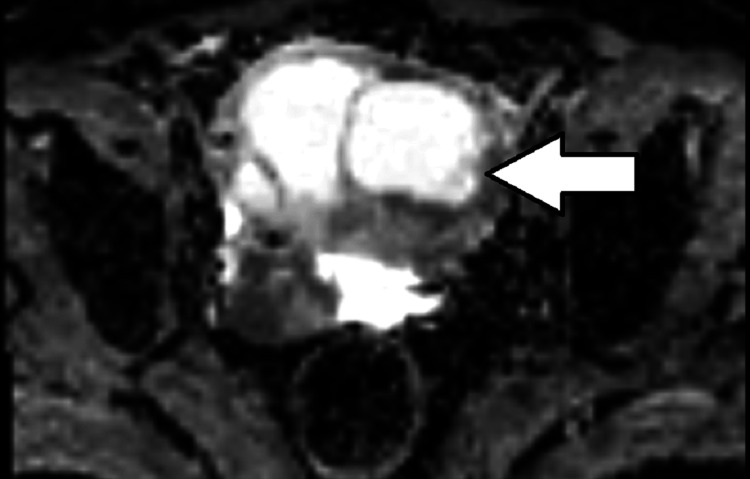
ADC (apparent diffusion coefficient) image of the septated cystic lesion of the cervix showing no restriction of diffusion.

A contrast-enhanced MRI of the pelvis was performed. A multiseptated cystic lesion, which appeared hypointense on T1-weighted images and hyperintense on T2-weighted images, measuring 5.5 x 3.8 cm, was seen. This lesion was located in the anterior cervical stroma, extending superiorly into the anterior myometrium of the uterus and laterally into the right broad ligament (Figures 2, 3). The lesion showed no solid components or calcification. There was no post-contrast enhancement (Figure 4). Diffusion-weighted imaging (Figure 5) showed a bright cystic lesion, and apparent diffusion coefficient images (Figure 6) were consistent with a cystic lesion with no restriction of diffusion. Hence, no obvious signs of infection or neoplastic lesions were observed. The junctional zone of the uterus appeared normal, with an appropriate endometrial thickness. Both ovaries were normal. The pelvic vasculature appeared normal in course and caliber, with normal flow voids. There was no evidence of pelvic lymphadenopathy or free fluid.

The findings suggested the diagnosis of a complex Nabothian cyst of the cervix.

This case report highlights the importance of using multiple imaging modalities to accurately diagnose the pathological condition in our patient. Notably, the patient was asymptomatic despite the presence of a large cystic lesion in the cervix. MRI facilitated the correct diagnosis of this incidental finding, thereby preventing unnecessary intervention. No treatment was required, and follow-up was suggested to the patient. If the cyst continues to grow, the patient may develop symptoms in the future. Consequently, the patient will be monitored for any changes in lesion morphology over time using appropriate imaging techniques, and necessary management will be considered as needed.

## Discussion

Nabothian cysts are common benign cystic lesions of the cervix, primarily occurring in women of reproductive age. These cysts are typically asymptomatic and are classified as simple mucous retention cysts [2]. Nabothian cysts are associated with chronic inflammation, which results in the proliferation of the squamous epithelium of the ectocervix over the columnar epithelium of the endocervix. Continuous glandular secretions contribute to the development of these retention cysts [4,5].

Nabothian cysts rarely increase in size and typically do not exceed 4 cm in their greatest dimension [1,6]. Large Nabothian cysts can mimic a malignant mass and may lead to symptoms such as abdominal pain and pelvic congestion [6]. Additionally, patients may experience vaginal bleeding and painful intercourse [5]. Due to the mass effect on the rectum, patients may have difficulty with defecation and may experience tenesmus [6].

These cysts typically do not require treatment due to their benign nature. However, treatment should be considered for symptomatic patients and those with suspicious morphology. For example, a rare condition known as adenoma malignum resembles large Nabothian cysts but involves an aggressive pathological process [1]. They appear as multicystic lesions containing a variable-sized solid component, extending from the endocervical gland to the deep stroma of the cervix. The solid component shows post-contrast enhancement, which differentiates it from Nabothian cysts. In such cases, the lesion should be treated with cryocautery, electrocautery, or complete excision, and a histopathological evaluation should be conducted [5].

The differential diagnosis for complex cystic lesions in the cervix includes endometriomas, leiomyomas, papillomas, adenoma malignum, and Nabothian cysts [1]. Most of these lesions present with symptoms such as abdominal discomfort, bleeding, or a protruding mass [6].

On ultrasound, complex Nabothian cysts appear as multiloculated cystic lesions with smooth, well-defined margins [7,8]. No internal vascularity is noted on color Doppler imaging [9]. On MRI, they present as septated cystic lesions exhibiting hypointensity on T1-weighted images and hyperintensity on T2-weighted images [10]. There is no wall enhancement observed with intravenous gadolinium [10]. However, capsular enhancement may occur if the cystic lesions become infected. Additionally, MRI can differentiate cystic mucinous neoplasms from Nabothian cysts due to variations in signal intensity within the cervical stroma on T2-weighted imaging [6]. Furthermore, benign lesions typically do not demonstrate wall enhancement with intravenous contrast [10]. Therefore, a small cystic lesion with well-defined, smooth boundaries and no enhancement is a characteristic finding of Nabothian cysts on MRI [7,9,10,11].

The presence of enhancement and solid components with irregular or ill-defined margins suggests a potential malignant etiology and necessitates further evaluation through histopathological analysis [11].

## Conclusions

This case report emphasizes the importance of utilizing various imaging modalities to accurately diagnose the pathological condition in our patient. Notably, our patient exhibited no symptoms despite the presence of a large cystic lesion in the cervix. The use of MRI led to the correct diagnosis of this incidental finding, thereby avoiding unnecessary intervention. If the cyst continues to grow, the patient may develop symptoms in the future. Therefore, the patient will be monitored for any changes in lesion morphology over time, with appropriate imaging modalities, and necessary management may be considered in the future.
